# Bone Health Management in the Continuum of Prostate Cancer Disease

**DOI:** 10.3390/cancers14174305

**Published:** 2022-09-02

**Authors:** Ettickan Boopathi, Ruth Birbe, Sunday A. Shoyele, Robert B. Den, Chellappagounder Thangavel

**Affiliations:** 1 Center for Translational Medicine, Department of Medicine, Thomas Jefferson University, Philadelphia, PA 19107, USA; 2 Laboratory Medicine, Department of Pathology, Cooper University Health Care, Camden, NJ 08103, USA; 3 Department of Pharmaceutical Sciences, Thomas Jefferson University, Philadelphia, PA 19107, USA; 4 Department of Radiation Oncology, Thomas Jefferson University, Philadelphia, PA 19107, USA; 5 Department of Dermatology, Thomas Jefferson University, Philadelphia, PA 19107, USA; 6 Department of Interdisciplinary Oncology, Department of Biochemistry & Molecular Biology, LSUHSC Stanley S. Scott Cancer Center, 1700 Tulane Ave, New Orleans, LA 70112, USA

**Keywords:** prostate cancer, androgen receptor, castration-resistant prostate cancer, bisphosphonate, taxane, radium-223, osteoblast, osteoclast, denosumab

## Abstract

**Simple Summary:**

In this review, we summarize the risk factors of prostate cancer (PCa), mechanism of PCa induced bone metastasis, current treatments for PCa induced bone metastasis, treatment induced side-effects, management of skeletal-related events and potential future therapeutic options for bone management in the continuum of PCa disease.

**Abstract:**

Prostate cancer (PCa) is the second-leading cause of cancer-related deaths in men. PCa cells require androgen receptor (AR) signaling for their growth and survival. Androgen deprivation therapy (ADT) is the preferred treatment for patients with locally advanced and metastatic PCa disease. Despite their initial response to androgen blockade, most patients eventually will develop metastatic castration-resistant prostate cancer (mCRPC). Bone metastases are common in men with mCRPC, occurring in 30% of patients within 2 years of castration resistance and in >90% of patients over the course of the disease. Patients with mCRPC-induced bone metastasis develop lesions throughout their skeleton; the 5-year survival rate for these patients is 47%. Bone-metastasis-induced early changes in the bone that proceed the osteoblastic response in the bone matrix are monitored and detected via modern magnetic resonance and PET/CT imaging technologies. Various treatment options, such as targeting osteolytic metastasis with bisphosphonates, prednisone, dexamethasone, denosumab, immunotherapy, external beam radiation therapy, radiopharmaceuticals, surgery, and pain medications are employed to treat prostate-cancer-induced bone metastasis and manage bone health. However, these diagnostics and treatment options are not very accurate nor efficient enough to treat bone metastases and manage bone health. In this review, we present the pathogenesis of PCa-induced bone metastasis, its deleterious impacts on vital organs, the impact of metastatic PCa on bone health, treatment interventions for bone metastasis and management of bone- and skeletal-related events, and possible current and future therapeutic options for bone management in the continuum of prostate cancer disease.

## 1. Introduction

Prostate cancer (PCa) is the second-leading cause of cancer-related deaths in men worldwide. The American Cancer Society estimates 268,490 new prostate cancer incidences in 2022, and that 34,500 men will die from this deadly disease. Risk factors for PCa include environmental factors, genetics, age, dietary habits, hormones, obesity, inflammation of the prostate, vasectomy, and microbiome infections. Age: Most prostate cancer is diagnosed in men 60 years of age and older. Based on previous studies, NCI Surveillance Epidemiology and End Results (SEER) estimates that the maximal incidence of PCa peaks between the ages of 65 and 74 (35.3%) [1,2,3]. Race: The incidence of PCa among U.S. men of all races is 156.0/100,000; Caucasians, 149.5/100,000; African Americans, 233.8/100,000; Asian and Pacific men, 88.3/100,000; Native Americans and Alaskans, 75.3/100,000; and Hispanic Americans, 107.4/100,000 [4,5]. Environmental factors: Men exposed to combustion byproducts and agricultural chemicals, such as pesticides, are at an increased risk of PCa development, and these pollutants are metabolized in the human body and impact on cellular metabolism by altering drug metabolizing enzymes (CYP450) and initiating PCa progression. Genetics: Sequencing and bioinformatic studies have revealed that there is an alternation in the DNA damage repair (DDR) pathways in prostate cancer. About 10% of primary tumors and 25% of metastatic tumors from prostate cancer harbor DDR defects with BRCA2 aberrations [6,7]. DNA repair genes, including ATM, CHEK2, ATM, PALB2, BRACA1, BRACA2, MLH1, MSH2, and MSH6, are frequently mutated in prostate cancer [8]. A previous study has also suggested the possible contribution of germline mutations in the DDR genes that contribute to metastatic prostate cancer [7]. In accordance with the earlier study [7], other groups have shown the contributions of germline mutations in the DDR genes to metastatic prostate cancer and about 8–16% of metastatic prostate cancer patients harbor germline deleterious mutations in the DDR genes [9,10].

**Figure 1:**
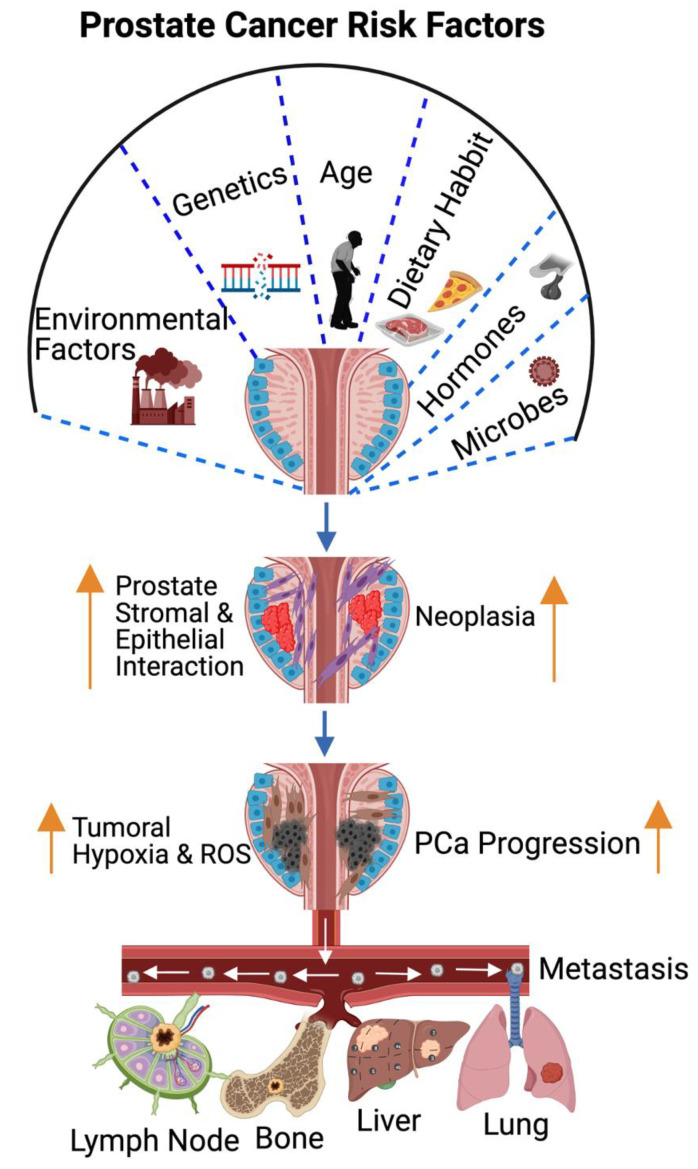
Risk factors in prostate cancer pathogenesis and metastasis. The well-studied prostate cancer risk factors are environmental factors, genetics, age, dietary habits, hormones, and microbial infection. The risk factors increase the ROS levels under tumoral hypoxia and promote stromal epithelial cell interaction and inflammatory signaling molecules that drive tumor initiation. ROS signaling promotes epithelial mesenchymal transition (EMT), and regional and distal metastasis (lymph node, bone, liver, and lung). The orange arrow indicates the increased or progression. This diagrammatic illustration was created with BioRender.com (accessed on 1 August 2022) agreement # OQ23NSL7AN.

Androgens play an essential role in the development of the prostate gland and are required for the maintenance of male physiology [11]. Androgens are secreted primarily at higher levels in gonads (testicles and ovaries) and adrenal glands. Conversion of the testosterone hormone to dihydrotestosterone (DHT) occurs in prostate, brain, and liver. Similar to the normal prostate, PCa cells require androgen for their growth and survival [12]. Androgen signaling via the androgen receptor plays a significant role in the pathogenies of PCa [13,14]. Since most prostate cancer cells require androgen-receptor signaling for their growth and survival, androgen deprivation therapy (ADT) is the preferred treatment for patients with locally advanced and metastatic disease that suppresses cancer cell growth. Despite the initial response to androgen blockade, most patients will eventually develop castration-resistant prostate cancer (CRPC) that leads to metastatic castration-resistant prostate cancer (mCRPC) [15,16,17,18]. Obesity: Previous studies have reported a positive association between obesity and PCa [19,20]. The impact of obesity on ADT outcome is limited and one of the studies has shown that obesity promotes CRPC, metastases, and PCa-specific mortality [21]. Insulin growth factor-1 axis, sex hormones, and adipokine signaling are the commonly proposed mechanisms that are possibly involved in connecting obesity and PCa [19,20,22,23,24]. Microbial infection: Microbial infection impacts androgen levels [25] and promotes PCa development. Additionally, meta-data analysis has identified the presence of *Escherichia* *Propionibacterium*, and *Pseudomonas* in PCa tissue [26], however, there are no further studies to support these findings. Previous studies have suggested that viral infections, such as human papilloma virus (HPV), herpes simplex virus type 2 (HSV-2), cytomegalovirus (CMV), human herpesvirus type 8 (HHV-8), Epstein-Barr virus (EBV), and polyomavirus BKV infections possibly promote PCa [27]. All these potential risk factors and the common metastatic sites of PCa are summarized in Figure 1.

The molecular pathogenesis of PCa is mediated through androgen-receptor (AR) signaling [28,29,30]. Most PCa deaths are attributed to castration-resistant-prostate-cancer (CRPC)-driven metastases. Most mCRPCs develop from androgen-sensitive PCa following androgen deprivation therapy (ADT). Based on clinical studies and genomic characterization, CRPC falls into two categories: (1) AR-dependent (AR mutation, amplification, and splice variants), (2) AR-independent (alterations in PTEN, TP53, and RB1 genes with neuroendocrine features). There are three ways by which PCa spreads to distal organs: (1) through the blood stream, (2) through the lymphatic system, and (3) through the wall into the abdominal and chest cavity. CRPC-disseminated tumor cells traveling through the blood stream are often attracted to vascular, osteoblastic, and hematopoietic niches in the bone and migrate to the lymph nodes, long bones, skull, lungs, and liver sites (Figure 1).

## 2. Bone Metastatic Castration-Resistant Prostate Cancer

The bone is one of the most-common metastatic sites for many solid tumors, including breast and prostate cancer. Bone metastases are common in men with mCRPC occurring in 30% of patients within 2 years of castration-resistant PCa and in >90% of patients over the course of the disease [31]. Patients with mCRPC-induced bone metastasis develop symptoms associated with skeletal-related events (SREs). The extent of bone involvement in mCRPC has been found to be associated with patient survival; the 5-year survival rate for these patients is 47% [32,33,34]. Previous studies have shown that both osteolytic, pro-osteoclastogenic factors, and osteoblastic components contribute to prostate cancer bone metastases [35]. The detection of bone metastases starts with Tc99m methylene diphosphate (MDP) bone/skeletal scintigraphy (SS), supported by plain film correlation, followed by magnetic resonance imaging (MRI), computerized tomography (CT), and position emission tomography (PET) to identify the early changes in the bone marrow that proceed the osteoblastic response in the bone matrix [36]. In addition, whole-body imaging modality is a bone scan currently being used to detect osseous metastases (a symptomatic bone metastases) induced by advanced prostate, lung and breast cancers [37]. In this review, we discuss: (1) PCa risk factors, the pathogenesis of PCa-induced bone metastasis, and its deleterious impacts on vital organs, (2) the impacts of metastatic PCa on bone health, (3) treatment interventions for bone metastatic prostate cancer and bone management, (4) the management of skeletal-related events, and (5) a summary, and future directions with the application of a multi-omics approach in PCa models, the identification and validation of drug targets, and management of bone health.

## 3. Pathogenesis of Prostate Cancer Bone Metastasis and Its Deleterious Impacts on Vital Organs

**Figure 2:**
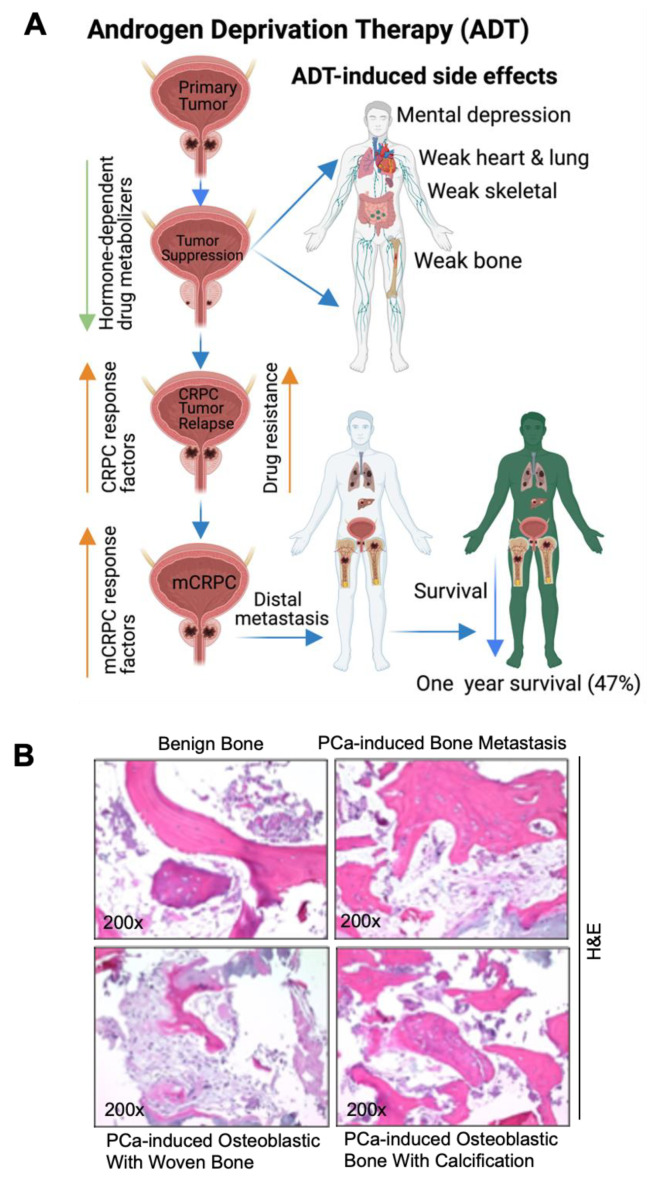
Pathogenesis of prostate cancer bone metastasis and its deleterious impacts in vital organs. (**A**) The schematic diagram shows that ADT suppresses PCa growth and simultaneously impairs hormone (androgen)-regulated drug metabolizers (cytochrome p450s). Additionally, ADT promotes mental depression, weakness in the bones, heart, lung, and skeletal muscles. ADT also promotes CRPC via AR-dependent and AR-independent mechanisms, drug resistance, and metastatic CRPC (mCRPC). mCRPC promotes local and distal metastasis, poor prognosis, and organ failure. (**B**) Bright field microscopic image of H&E-stained biopsy samples from (1) a male benign bone PCa (top left), (2) prostate-cancer-induced bone metastasis (top right), (3) prostate-cancer-induced bone metastasis with osteoblastic with woven bone (bottom left), (4) prostate-cancer-induced bone metastasis with osteoblastic bone with calcification (bottom right), magnification 200×. The up-arrow indicates increase and the down arrow indicates decrease. This diagrammatic illustration was created with BioRender.com agreement # CY23NSMIZH.

PCa is a largely hormone-driven cancer, so androgen deprivation therapy (ADT) is increasingly used for the treatment of hormone-resistant PCa. However, ADT is possibly associated with numerous side effects, including an increased therapy-related sarcopenic obesity, mental depression/health, weaker lung and heart muscles, bone weakness, bone fracture, cardiovascular mortality, and altered expressions of hormone-dependent drug-metabolizing enzymes (human cytochrome p450). The deregulation of CYP450 enzymes leads to altered drug metabolism, drug resistance [38], CRPC and mCRPC development. The androgen receptor (AR) signal is considered to be the key driver of CRPC and mCRPC [39]. Approximately 80–90% of men with advanced PCa will develop bone metastasis, and most CRPC promotes bone metastasis [40,41]. In addition, a growing body of literature suggests that mixed-lineage protein kinase 3 (MLK3) signaling initiates WNT signaling and in turn WNT signaling promotes bone metastases in ADT-resistant PCa cells [42,43]. Advanced PCa-induced metastasis leads to organ failure and acute respiratory disorders [44]; the follow-up from the PCa initiation and the 1-, 3-, and 5-year survival rates of metastatic patients are 89.1%, 76.9% and 49.8%, respectively, however the 1-year survival rate of metastatic PCa without bone incidence is 87% and with bone incidence is 47% [32,33,34,45,46] (Figure 2A). While breast and other cancers form osteolytic lesions, bone metastasis of prostate cancer is characterized by an osteoblastic appearance which is caused by the deregulation of bone resorption and bone formation [47,48,49,50]. We also identified osteoblastic bone metastasis with calcification from deidentified PCa-induced bone metastatic biopsies via H&E staining (bright field microscopic view, (Figure 2B)) and these results correlated with earlier reports [51,52]. Deidentified clinical samples were obtained from Thomas Jefferson University Hospital in accordance with Institutional Review Board Standards and in compliance with federal regulations governing research on deidentified specimens as described [53]. Bone metastasis causes strong bone pain, compression of the spinal cord, hypercalcemia, and increases mortality.

## 4. Bone Stromal and Prostate Cancer Cell Interaction Promotes Bone Metastasis

Changes in the stromal tissue associated with tumors and changes in the tumor cells themselves contribute to the ability of metastatic cancer cells to escape from tumor sites. Fibroblasts are associated with solid tumors or cancer-associated fibroblasts (CAFs) frequently acquire an activated myofibroblast phenotype in response to growth factors and the tumor microenvironment. The activated fibroblast produces elevated levels of matrix metalloproteinases (MMPs) and remodels the extracellular matrix (ECM) of tumors [54]. Cancer cells and the activated fibroblasts secrete high levels of VEGF-growth factor and the CXCL-family of chemokines, which recruits leukocytes and endothelial cells into the tumor microenvironment [54]. Tissue remodeling creates a microenvironment permissive of cancer cell escape and a tumor microenvironment (TME) with changes in the genetics of transformed cells possibly promotes metastasis. PCa patients often develop skeletal metastases; the establishment of metastases within the bone is a complex-multistep process, including colonization, dormancy, reactivation of dormant cancer cells, development, and reconstruction. Initially, the circulating cancer cells enter into the bone marrow, adapt to the bone microenvironment and remain in dormant status. The dormant cancer cells subsequently get reactivated to an active proliferative state and change the original/native bone structure and function [55,56].

## 5. Mechanisms of Prostate-Cancer-Induced Bone Metastasis

Tumor-escaped PCa cells can reach the bone by traveling from local to the regional sites via the blood stream and establish molecular interactions with bone stromal cells, osteoblasts, osteoclast cells, and promote bone metastases [54,57,58]. PCa and bone resident cells interact with each other in response to various growth factor signals [54,59]. Bone marrow contains the hematopoietic stem cells (HSCs), osteoblasts, osteoclasts, and mesenchymal stem cells (MSCs). Previous studies have demonstrated that PCa cells influence bone resorption by osteoclasts and bone formation by osteoblasts by secreting monoamine oxidase A and eventually promote cancer cell progression.

**Figure 3:**
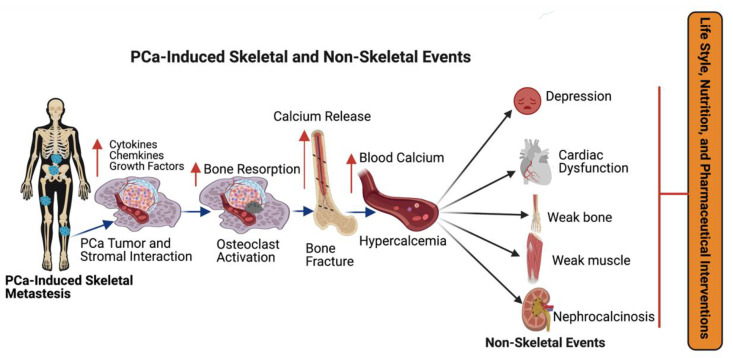
The impact of metastatic prostate cancer on bone health and bone-metastasis-induced skeletal and non-skeletal events. Prostate cancer often promotes bone metastasis (skeletal metastasis). The prostate cancer cells in the bone interact with bone stromal cells and promote the expression of cytokines, chemokines, growth factors, and osteoclast cell activation. Osteoclast activation initiates bone fracture and calcium release into the blood stream. Excess calcium in the blood stream promotes mental depression, cardiac dysfunction, bone, and muscle weakness, and nephrocalcinosis (kidney disorder) and skeletal and non-skeletal events are managed with lifestyle, nutritional and pharmaceutical interventions. This diagrammatic illustration was created with BioRender.com agreement # JO23NSOQ0.

Hematopoietic stem cells (HSCs) serve as a foothold for PCa during their metastases to bone and mesenchymal stem cells (MSCs) and are found to increase the metastatic ability of the PCa cells by modulating AR signaling. PCa cells secrete active molecules that promote osteoblast differentiation, proliferation, including endothelin-1, and transforming growth factor β (TGF-β) [60]. In turn, enhanced osteoblast activity drives tumor progression by releasing interleukins 6 and 8 (IL-6 and IL-8) and insulin growth factor (IGF-1) [61]. PCa cells express angiogenic and bone-resorbing factors that induce cancer cell growth within the bone microenvironment [54,58]. Additionally, prostate cancer cells interact with bone stromal cells and promote the expression of cytokines, chemokines, and growth factors (Figure 3). Most importantly, growth factors, such as transforming growth factor, fibroblast growth factors, platelet-derived growth factors (PDGF), and bone morphogenetic proteins (BMP) are found in the bone matrix [62,63,64] and all these growth factors have been reported to participate in PCa metastasis. Osteoclasts are capable of degrading bone and tissue and releasing minerals, calcium, magnesium, and phosphate, and also degrading bone collagens into the blood stream and promoting hypercalcemia [65]. Bone calcium is removed from the bone via an osteolytic process and this causes bones to become weaker, leading to bone fractures and osteoporosis [66]. Continuous hypercalcemia promotes mental depression, weaker heart muscles, weaker bones (Figure 3), and nephrocalcinosis/kidney disorder [67]. Advanced prostate-cancer-induced bone metastases and bone-metastases-induced skeletal events (SREs) can be managed by lifestyle, nutritional, and pharmaceutical interventions as shown in Figure 3.

## 6. Therapeutic Options for PCa-Induced Bone Metastases and Bone Management

**Figure 4:**
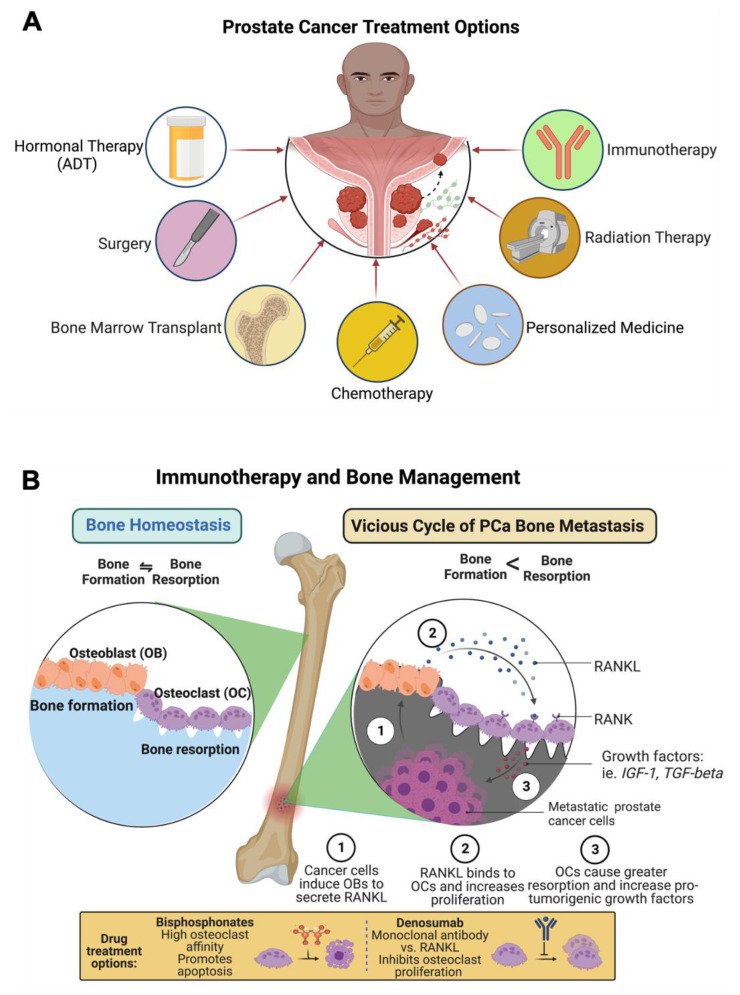
Treatments options for PCa-induced bone metastasis and bone management. (**A**) Patients with PCa-induced bone metastasis are treated with surgery, hormonal therapy (androgen deprivation therapy, ADT), bone marrow transplant, chemotherapy (taxanes, bisphosphonate), anti-androgen therapy (enzalutamide), personalized medicine (T-cell therapy), radiation therapy (radium-223), and immunotherapy (denosumab). (**B**) Prostate cancer treatment, including immunotherapy and bone management, is presented in the figure as a schematic diagram. The diagram shows that bisphosphonate reduces osteoclast numbers by promoting apoptosis. Anti-RANKL monoclonal antibody denosumab therapy inhibits the development and activity of osteoclasts, followed by the suppression of bone resorption. This diagrammatic illustration was created with BioRender.com agreement # ET23NSSNDQ and TC23NSPRCC.

The treatment options for metastatic prostate cancer are androgen deprivation therapy (ADT), surgery, bone marrow cell transplants, chemotherapy (taxanes and bisphosphonates), personalized medicine (T-cell therapy), radiotherapy or radiopharmaceuticals or a combination of chemo-radio therapy and immunotherapy (Figure 4A).

## 7. Androgen Deprivation Therapy (ADT) for CRPC-Induced Bone Metastasis

As in the normal prostate, PCa cells require androgens for their growth and development [12]. The requirement of androgens is exploited using ADT, also termed therapeutic castration as a first-line therapy for metastatic or recurrent PCa patients. ADT by means of surgical castration or pharmacological therapy reduces serum testosterone levels by 90–95%. Surgical castration, also called orchiectomy (removal of testicles), or injecting the luteinizing hormone-releasing hormone (LHRH) agonists (also called LHRH analogs) or Gonadotropin-releasing hormone (GnRH agonists) lowers serum testosterone levels by 90–95%. The injection of LHRH agonists, also called GnRH agonists (e.g., leuprorelin (Lupron); or goserelin (Zoladex)), and the injection of an LHRH antagonist, (e.g., degarelix (Firmagon); or relugolix (Orgovyx)) lowers testosterone levels. ADT has excellent initial responses; however, in most cases it results in PCa disease relapse within a few years of treatment because of the alternative mechanisms of AR signaling which promotes CRPC. The options for CRPC therapy are antiandrogen treatment, taxane-based chemotherapies, sipuleucel-T (Provenge) vaccine, or radium-223. First-generation anti-androgens, such as flutamide and bicalutamide, exclusively target AR translocation into the nucleus by the competitive inhibition of androgen binding to AR [68,69] and prevention of AR downstream signaling. Second-generation antiandrogens, such as enzalutamide, apalutamide, and darolutamide, prevent AR nuclear translocation to a greater extent than first-generation agents [70,71,72,73,74,75,76]. CRPC-induced bone metastases are also treated with a combination of chemotherapy and anti-androgens. Docetaxel or enzalutamide fused with ADT are effective therapies in bone metastatic prostate cancer patients [77]. Additionally, PCa patients with bone metastasis are also treated with docetaxel, abiraterone acetate, enzalutamide, or apalutamide in combination with ADT [78,79,80], to reduce the tumor burden and also to inhibit skeletal-related events, such as pathological bone fracture, bone resorption, spinal cord compression, surgery to the bone, and radiation to the bone. The adverse effects of hormonal therapy include: impaired drug metabolism, reduced bone mineral density, weight gain, decreased muscle mass, diabetics, libido decrease, sexual dysfunction, hot flashes, reduced testicle size, cardiovascular dysfunction, deep vein thrombosis, erectile dysfunction, depression, and reduced cognition [81,82].

## 8. Surgery

Multiple interventional approaches are used in bone management in advanced prostate-cancer-induced bone metastasis. (1) Surgery (stabilization of bone), which involves the removal of tumors from the bone and stabilizing the breaking bone with metal plates, screws, and nails. These processes will relieve pain and improve bone function. Following stabilization, often radiation therapy will be advised to restrict tumor growth. (2) Following surgery, the injection of cement is carried out to reinforce the metal plates in pelvic and spine bones, and this protocol will reduce pain. (3) Surgery to fix fractured bones: in this process, the bones are stabilized with metal plates and screws in the fractured area. Prostate bone metastases are often osteoblastic, and other cancer skeletal metastases are a combination of osteoblastic and osteoclastic. The treatment for PCa-induced skeletal metastases includes orthopedic surgery to relieve spinal compression and pain [83,84].

## 9. Chemotherapy

Katsumi et al. in 2020, summarized their view on a bone-targeted drug-delivery system and strategies for the treatment of bone metastasis [85]. Several articles have been published on the application of targeted and conventional drug delivery for bone metastases. Targeted drug-delivery systems utilize tetracycline, carboxylic acids, bisphosphonate, amino acids, and aptamers for bone metastases and bone management at clinical and preclinical levels. Many studies have postulated that pro-anabolic and anti-catabolic actions of tetracyclines on MMPs and bone resorption [86]. Additionally, they inhibit pro-inflammatory cytokines and other inflammatory agents. Tetracyclines are aided by osteoblast stimulation, MMP inhibition, and attenuation of bone resorption. They reduce pathologically elevated levels of MMPs [87], pro-inflammatory cytokines, and other inflammatory agents. However, conventional drug delivery routes, such as oral and infectible routes have limitations, such as limited drug delivery in the bone and drug efficacy, the accumulation of drugs in non-targeted organs and the promotion of toxicity.

At present, bisphosphonates (BPs) are the most widely used drugs in the prevention of skeletal-related events in patients with breast and prostate cancers [88,89]. BPs, such as Actonel, Fosamax, Boniva, Reclast, and Aredia are the first successful pharmacological agents employed for the treatment of prostate cancer bone metastasis. Among these agents, Reclast stands first in its overall rating in the management of skeletal-related events. BPs have the ability to restrict skeletal-related events, such as hip and spinal fractures in osteoporosis during metastatic prostate cancer treatment, as shown in Figure 4B [85]. Bisphosphonates inhibit osteoclasts, cancer growth and strengthen bones, reduce fractures, and maintain blood calcium levels [90,91,92,93]. BPs inhibit osteoclast-mediated bone resorption by inhibiting a ruffled border, where the osteoclasts adhere to the bone surface [94,95,96]. In addition to these, BPs restricts the osteoclast number and their function by suppressing osteoclast progenitor development and inducing osteoclast apoptosis [97]. Structurally, BPs contain two phosphate groups which are linked by esterification, and these are stable derivatives of inorganic pyrophosphate [98]. Bisphosphonates are of two types: (1) Nitrogen-containing, and (2) Non-nitrogen BPs. These two classes of BPs are metabolized differently. Following the osteoclast-mediated uptake of BPs from the bone mineral surface, the non-nitrogen-containing BPs (etidronate, clodronate, and tiludronate) become incorporated into molecules of newly formed adenosine triphosphate (ATP) because of the close similarity to inorganic pyrophosphate and these nonhydrolyzable ATP analogues inhibit multiple ATP–dependent cellular processes, leading to osteoclast apoptosis [99,100]. The nitrogen-containing BPs (N-BPS, e.g., pamidronate, alendronate, ibandronate, risedronate, and zoledronate) inhibit farnesyl diphosphate synthase, a key regulatory enzyme in the mevalonic acid pathway which is critical for the production of cholesterol, other sterols, and isoprenoid lipids in the mevalonate pathway [101,102,103]. BPs are well-tolerated by most patients; however, the efficacy and safety of BPs varies among patients [104]. Approximately, 5–10% patients fail to respond to BP therapy [105,106]. In addition, intravenous BP treatment in some patients have caused side effects, such as atrial fibrillation [107], acute phase response [108] in the muscle, joint pain, kidney disease, heart burns, and lowering calcium levels among prostate cancer patients [109,110].

Taxanes are a class of diterpenes that were originally discovered from plants belonging to the Taxus genus. Paclitaxel, docetaxel and cabazitaxel are the commonly used taxanes in clinical practice [111]. The therapeutic value of docetaxel in patients with CRPC was established by two lead clinical trials, TAX327 [112] and Southwest Oncology Group (SWOG) [113], and the survival benefits of docetaxel from these two pivotal clinical trials were published in 2004. The first of these trials, TAX327, was a randomized Phase III trial performed in 1006 patients [112]. Docetaxel-based treatment significantly conferred palliative relief and improved the overall survival benefit compared to control-treated patients [111]. However, the Docetaxel-based treatment ultimately failed with the majority of the patient’s developing resistance. The FDA-approved Cabazitaxel is a next-generation semisynthetic taxane chemotherapeutic agent which was shown to be effective in the Docetaxel-resistant CRPC landscape [114]. Taxanes have the ability to prolong survival in patients with metastatic CRPC and hormone-sensitive prostate cancer [111,113,115,116]. The mechanism of action of taxanes has been well-established as a microtubule-targeting agent. Paclitaxel and docetaxel prevent the depolymerization of microtubules and thus prevents the progression of cancer cells through the G2 and M-phases of the cell cycle [117]. Taxanes bind to tubulin and stabilize microtubules which leads to an enhanced polymerization of microtubules and prevents disaggregation of the spindle apparatus [117]. The stabilization of microtubules results in cell cycle arrest and apoptosis [117]. The main side effects of the use of taxanes include hearing loss, skin reactions, edema, and neurotoxicity [118,119].

## 10. Bone Marrow Cell Therapy for Metastatic Prostate Cancer

There is a critical need to develop bone marrow cell therapy in bone management that targets the bone, the primary metastatic site for prostate cancer. Unfortunately, very little information is available in the literature regarding bone marrow cell therapy that targets metastatic prostate cancer. Wang and Thompson, 2008 summarized the importance of developing gene-modified bone marrow cell therapy for advanced prostate cancer [120]. A previous study has demonstrated that IL-12-gene-modified bone marrow cell therapy suppressed the development of experimental prostate cancer metastasis in a preclinical mouse model [121]. The administration of IL-12-gene-modified adult bone marrow cells to the mice induced significant anti-metastatic effects in the bone and lungs [121]. However, the translational of bone-marrow-based IL-12 therapy to clinical practice ultimately failed, with the majority of the patients developing metastatic prostatic cancer. Recently, Sidney Kimmel Comprehensive Cancer Center at Johns Hopkins has initiated a pilot clinical trial (NCT02995330) on Sex-linked Mismatched Allogeneic Bone Marrow Transplantation for Men with Metastatic Castration-Resistant Prostate Cancer (mCRPC). Briefly, progressive mCRPC patients underwent ADT or ADT with docetaxel. Patients who had an identified related female donor (mother, sister, daughter, granddaughter, or niece) received bone marrow transplants followed by post-Cytoxan (PT/Cy) and testosterone treatment.

## 11. Personalized Medicine/Cancer Vaccine

Autologous immunotherapy for castration-induced prostate cancer [122,123] is called “sipuleucel-T vaccine therapy”. This vaccine was developed from men who did not respond to hormone therapy. This vaccine was made from a patient’s white blood cells (dendric cells) and these cells were mixed with protein prostatic acid phosphatase (PAP) from a PCa cell and immune-tuned dendric cells injected into the same patient. This process is repeated two to three times at intervals of two weeks. Similarly, peripheral-blood mononuclear cells (PBMCs) and antigen-presenting cells (APCs) activated with a fusion protein (PA2024) possessing prostate antigen prostatic acid phosphatase (PAP) fused to macrophage-colony-stimulating factor (GM-CSF) are also used in the prostate cancer vaccine [124,125].

## 12. Immunotherapy

Morgenroth et al. (2007) [4] developed the functional chimeric T-cell receptor (CTCR) against prostate stem cell antigens to treat and manage prostate cancer (chimeric alpha-PSCA-beta2/CD3zeta-TCR) [4,126]. Immune checkpoint inhibitors are made to destroy the cancer cells at cell cycle checkpoints, and these revolutionary therapies stimulate T cells to preferentially target prostate cancer cells as a single agent or in combination with other agents [127,128,129,130,131,132,133,134,135,136,137]. Immune checkpoint therapies have shown great efficacy in many tumor models, and however CRPC-induced bone metastasis has a sub-optimal response to them [138], suggesting some immunological niche in the bone environment. Jiao et al. discovered distinct immune cell subsets that are responsible for the sub-optimal response of CRPC-induced bone metastases to immune checkpoint therapies [138]. Bone homoeostasis is regulated by the dynamic well-balanced actions of osteoclasts, osteoblasts, and osteocytes. Osteoclasts are differentiated from osteoclast precursor cells in the presence of certain essential factors: the receptor activator of nuclear factor-kB (NF-κB) ligand (RANKL), a tumor necrosis factor (TNF) family of cytokines, the macrophage/monocyte colony-stimulating factor (M-CSF), prostaglandin-E2 and PTHrP (Parathyroid-hormone-related protein) [54,139,140,141,142]. However, osteoblast activity has been shown to be deregulated in cancer cell metastasis. The cancer cells imbalance the osteoblast and osteoclast populations and promote bone degradation (osteoclast metastases). After reaching the bone, cancer cells secrete PTHrP, which triggers the osteoblast cells to release RANKL and simultaneously inhibit RANKL antagonist osteoprotegerin (OPG) with other growth factors, including TGF-β, and endothelin-1 (ET0-1). RANKL overexpression leads to osteoclast activation and bone matrix degradation. Recent advancements in immunotherapy-formulated monoclonal antibody (denosumab) (Figure 4B) therapy against RANKL show that it prevents the binding of the RANKL receptor to RANKL and it inhibits SREs as described in clinical trial # NCT00089791. In another clinical trial, NCT00321464, denosumab was compared with zoledronic acid and the data on comparative studies confirmed that both the agents were effective in SREs and denosumab was more efficient than zoledronic acid [143]. The common side effects noted with denosumab administration include: skin infections, back, arm, and neck pain, constipation, urinary tract infections, and rashes; less common and rare side effects noted are low blood calcium, and osteoporosis of the upper jaw and fractures [144,145,146].

## 13. Radiation Therapy

Generally, PCa cells migrate to osteoblastic lesions of the bone and promote pain, fracture, and bone dysfunction, as well as bone formation and bone damage within the metastatic regions [147,148,149,150]. Osteoblastic lesions in the bone are the frequent metastatic sites for PCa, and these lesions frequently cause bone pain, fractures, and bone dysfunction. Radiation therapy, as a monotherapy or as an adjunct to other treatments, plays a significant role in the treatment of skeletal bone metastasis [151,152]. Radiation therapy can provide significant palliation of symptoms for patients and can prevent the morbidity of bone metastasis. It is highly effective in controlling pain. In the treatment of bone metastases, external-beam radiation and systemic radiotherapy with radioisotopes are used and with external-beam radiation, pain relief is obtained in 50–80% of patients [153,154,155,156,157,158]. External-beam radiation therapy employs three-dimensional conformational radiation therapy (3D-CRT) or intensity-modulated radiation therapy (IMRT) or stereotactic body radiation therapy (SBRT) to deliver the radiation dose to the tumor.

In addition, radiopharmaceuticals are used to treat PCa-induced bone metastasis. Both α- and β-particles emitting radiopharmaceuticals deliver radiation directly and specifically to cancer cells. α-(Radium 223) and β-particles (strontium-89) (Sr-89) chloride and samarium-153-emitting radiopharmaceuticals are used for the palliation of pain caused by bone metastases from PCa [159,160,161,162,163,164,165,166,167]. Radiopharmaceuticals have been underutilized in clinical practice because of their notable side effects which include myelosuppression and mutagenesis [168]. Radium 223 has a high affinity for the bone matrix and acts as a calcium mimetic by forming complexes with the bone mineral hydroxyapatite in areas of increased bone turnover [169,170,171]. Radium-223 has also been used in the EORTC Genito-Urinary Cancers Groups phase III clinical trial with the combination of Enzalutamide, and Lutetium 177-PSMA-617 [170,172]. Bone metastases are hard to remove completely, in such situations the innovative Lutetium-177-PSMA-617 is employed, and is also under clinical usage for mCRPC (NCT03511664).

Osteoclast differentiation has one of the most major impacts on bone lysis and excess calcium release, and the application of Radium-223 alone or in combination with abiraterone has been shown to inhibit osteoclast differentiation in vitro and in patients with bone metastases. Men who received Radium-223 lived longer without skeletal events than those who received the placebo, however there were some limitations noted by the trial leader Dr. Christopher Parker (The Royal Marsden National Health Service Foundation Trust and Institute of Cancer Research (Sutton, UK)) that the trial did not include men with visceral metastases, which are present in 25% of CRPC [170,173,174,175,176]. Radium-223 increases the overall survival of CRPC patients with bone metastases and reduces death by 30%. [170]. However, Radium-223 and Lutetium-177 PSMA promote common side effects, such as nausea, vomiting, dry mouth, headache, diarrhea, peripheral edema, and low blood counts in 10% of patients and cause less-common side effects, such as kidney failure, redness, pain, swelling of injected sites, and dehydration in 1–5% patients [177,178]. The α-particle emitters deliver a more-localized radiation with very short ranges, and they have higher mutagenic effects by inducing double-stranded DNA breaks but far less myelosuppression to the bone marrow compared with β-particle emitters.

## 14. Advanced Prostate Cancer and Bone-Metastases-Induced Skeletal-Related Events

Men with advance prostate cancer are at the risk of bone metastases which lead to several potential complications, including skeletal-related events (SREs). The major skeletal complications associated with bone metastasis include cancer-induced pain, hypercalcemia, pathological bone fractures, and metastatic spinal cord compression [179]. In addition to the significant effects on measures of health-related quality of life in men with advanced prostate cancer and bone metastases, SREs also have an important economic consequence. For example, orthopedic or spinal surgery and other treatments for SREs add to the already-significant cost burden of advanced cancer [180].

## 15. Management of Skeletal-Related Events during Advanced Prostate Cancer Treatment

**Figure 5:**
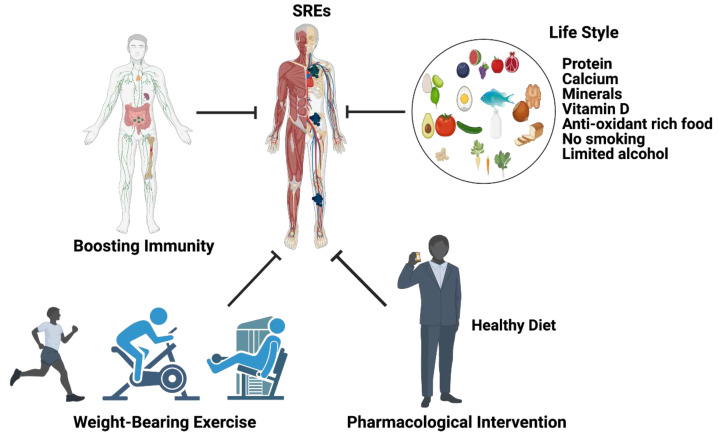
Management of PCa bone metastasis induced skeletal-related events (SREs). The cartoon illustrates that changing your lifestyle to a healthy diet with no alcohol and no smoking limits the development of SREs. Weight-bearing exercise for 40 h per week in SRE patients can help to restrict SREs. Boosting immunity also decreases SREs. Pharmacological interventions also help to limit SREs. This diagrammatic illustration was created with BioRender.com agreement # LO23NSQII5.

Following a good lifestyle, balanced healthy diets, pharmacological interventions, and boosting immune systems will possibly limit SREs during bone metastasis. The following recommendations are from the endocrine society clinical practice for men: 1000 mg calcium/day for those aged between 19–70 and 1200 calcium mg/day for men aged 71 years or older could reduce SREs [96,181]. In addition, the endocrine society clinical practice for men recommends that men with SREs are required to take 600 international units (IU) of vitamin D for those up to age 70 and 800 IU of vitamin D for those aged 71 years or older. In addition to this, SRE patients are supposed to reduce their alcohol consumption from three units of alcohol to one unit (one unit = 10 of pure alcohol) and no smoking is recommended. The American Urology Association 2020 recommends the application of bone-protective denosumab, a RANKL inhibitor or zoledronic acid, a bisphosphonate [94] as a pharmacological intervention (as described earlier) along with calcium and vitamin D supplementation to prevent SREs (Figure 5).

## 16. Summary and Future Directions

Prostate-cancer-induced bone metastasis is a complex entity with a variety of risk factors, including age, genetics, environmental factors, dietary habit, and microbial infections (Figure 1). PCa-induced bone tumors are diagnosed with ultrasound, computerized tomography (CT) magnetic resonance imaging (MRI), position emission tomography (PET), and bone scan index (BSI) [182]. BSI is used to determine the efficacy of drug treatment and to quantify the cancer burden within the bone [183,184,185,186]. PCa-induced bone metastasis is a complex process that involves several steps, including metastatic colonization of circulating tumor cells (CTCs) [187]; when these cells become quiescent (dormant), they are referred to as disseminated tumor cells (DTCs). The quality and quantity of CTCs can be used as markers for prostate cancer detection. Additionally, the AR-variant-7 (AR-V7) also functions as a PCa metastasis marker in CRPC [188]. Prostate cancer bone metastasis can be osteoblastic or osteoclastic or both [189]. Osteoblastic cells express biomarkers, including ET-1, BMPs, and Wnt, and promote aberrant bone deposition. Accordingly, osteoclastic cells express PTHrP, IL-6, IL-11, TGF-β, TNF-alpha, and MMPs [189,190,191,192,193,194], and all of these factors interact with the bone microenvironment and promote bone resorption and bone structural modifications, targeting the entire skeletal system and targeting specific cellular locations (osteoblast and osteolytic cells or both) [195].

Earlier studies have demonstrated that both osteolytic, pro-osteoclastogenic factors and osteoblastic components contribute to prostate cancer bone metastases. Bone metastases are known to cause bone pain, compression of the spinal cord, hypercalcemia, and increase mortality. Osteoclasts are capable of degrading the bones and releasing excess calcium into blood and promoting hypercalcemia, promoting mental depression, weaker heart muscles, weaker bones, and nephrocalcinosis (kidney disorder). The first line of treatment for bone metastatic prostate cancer is androgen deprivation therapy, which is very effective for the initial years. Additionally, PCa patients with bone metastasis are treated with first- and second-generation drugs, including docetaxel, abiraterone acetate, enzalutamide, or apalutamide in combination with ADT to reduce the tumor burden and also to inhibit skeletal-related events, such as bone fracture, and osteolysis. Hormonal therapy promotes side-effects, such as impaired drug metabolism, reduced bone mineral density, weight gain, decreased muscle mass, diabetics, libido decrease, sexual dysfunction, hot flashes, reduced testicle size, cardiovascular dysfunction, deep vein thrombosis, erectile dysfunction, depression, and reduced cognition. Bones are managed via surgical stabilization, which improves pain and bone physiology following radiotherapy. The application of chemotherapy via a targeted drug-delivery system, including tetracycline, carboxylic acids, bisphosphonate, amino acids, and aptamers for bone metastases are used for bone management during treatment. Currently, bisphosphonates (BPs) are employed to prevent skeletal-related events in patients with breast and prostate cancers. Recently diterpenes (taxanes) are employed in the management of CRPC and mCRPC, however these agents cause side effects, such as hearing impairment, skin reactions, edema, and neurotoxicity. Bone marrow cell therapies for mCRPC are still in developmental stages. Similarly, personalized medicine/cancer vaccines are developed from the same patient’s dendric cells and mixed with protein prostatic acid phosphatase (PAP) from a PCa cell and injected into the same patient, however these treatment options need further improvement. Additionally, immune checkpoint therapies have shown great efficacy in many tumor models, and however CRPC-induced bone metastases have a sub-optimal response to them, suggesting some immunological niche in the bone microenvironment. Radiotherapies, including α-(Radium 223) and β-particles (strontium-89) (Sr-89) chloride and samarium-153-emitting radiopharmaceuticals are efficient treatment options that are effective in controlling bone pain management. Bone-metastases-induced skeletal-related events are managed via a good lifestyle, balanced diets, pharmacological interventions, and boosting the immune system will possibly limit SREs during bone metastasis. However, these treatment options are not efficient, because ADT increases faster drug clearance via CYP450 enzymes that reduce drug efficacy. Together, all these events reduce the availability of drugs in the target cells. Recent advancements in drug delivery have increased drug efficacy by using nanoparticle delivery. The application of nanoparticle-mediated drug delivery will be an ideal system to improve drug stability and efficacy.

**Figure 6:**
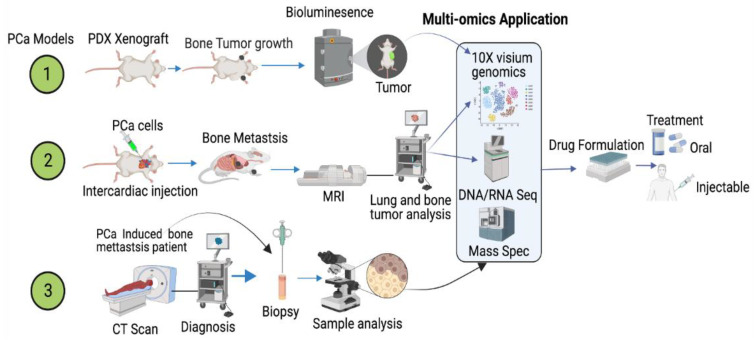
Multi-omics approach in the identification of potential therapeutic drug targets for prostate cancer. Schematics depict the application of a multi-omics approach in the identification of drug targets. Samples collected from patient-derived (bone mets) xenograft (PDX) (1), PCa-induced lung metastasis (2), and PCa bone mets (3) are subjected to multi-omics analysis. This diagrammatic illustration was created with BioRender.com agreement # JU23NSRKRQ.

**Figure 7:**
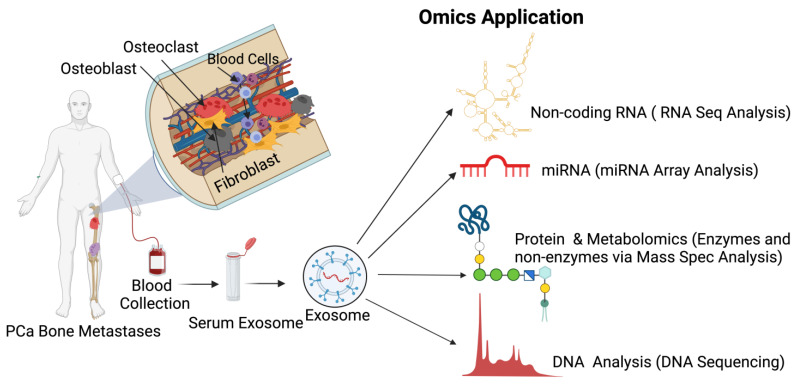
Exosomes as a diagnostic tool to identify biomarkers in prostate cancer. Exosomes are non-invasive biomarkers and hold great potential for the diagnosis of prostate cancer. Multi-omics approaches to exosome analysis are summarized in the figure as a schematic diagram. Exosomes are isolated form the blood samples collected from prostate-cancer-induced bone metastatic patients. The non-coding RNA, micro-RNA, proteins, and cell-free DNAs of the exosomes can be identified by RNA, DNA sequencing, 10× spatial genomics, and mass spectrometry. This diagrammatic illustration was created with BioRender.com agreement # IB23NST298.

However, all these technologies and biomarkers are not efficient enough to predict druggable targets to protect bone health while treating PCa-induced bone metastasis. Therefore, the application of cutting-edge multi-omics technologies is warranted on preclinical, patient, and patient-derived models to delineate and define accurate targets to reduce mortality among PCa patients (Figure 6). In addition to these cancer models, recent advancements in the application of extracellular vesicles/exosomes as a diagnostic tool to detect biomarkers for various diseases, including cancer, are clinically warranted [196,197,198,199,200]. Prostate cancer cells preferentially reach the skeleton, interact with osteoblasts, osteoclasts and activated bone fibroblasts, and fibroblasts, osteoblasts, and osteoclast cells excrete extracellular vehicles (exosomes). Extracellular vesicles are signaling molecules, which function as a cargo and they are rich in long non-coding and coding RNAs, enzymes, proteins, amino acids, microRNA, and cell free DNAs. The application of multi-omics technologies, such as total RNA sequencing, single cell RNA sequencing, 10× spatial genomics, whole exome sequencing, metabolomics, and mass spectrometry will identify stage-specific biomarkers which will reflect and present the disease state and the bone health and treatment options. This approach will help to identify biomarkers for early and late PCa-induced metastasis and bone management during treatment (Figure 7).
